# Epidemiological profile of patients attending the maxillofacial prosthodontics unit at Ibn Sina University Hospital in Rabat, Morocco: a cross-sectional study

**DOI:** 10.11604/pamj.2024.48.21.40925

**Published:** 2024-05-24

**Authors:** Mohamed Azhari, Abdoulmajid Habibou, Oussama Bentahar

**Affiliations:** 1 Faculty of Medicine, Pharmacy and Dental Medicine of Fez, University Sidi Mohamed Ben Abdallah, Fez, Morocco,; 2 Military Hospital, Niamey, Niger,; 3 Head of Maxillo-Facial Prosthodontics Unit, Ibn Sina University Hospital, Rabat, Morocco

**Keywords:** Epidemiology, maxillofacial prosthesis, oral rehabilitation

## Abstract

**Introduction:**

the present study aimed to establish an epidemiological profile of patients consulting the unit of maxillofacial prosthodontics in Rabat. Results deriving from this study will help enhance the quality of patient care in our center and can also serve as a comparison tool with other maxillofacial teams around the world.

**Methods:**

during 11 months all patients consulting the unit of maxillofacial prosthodontics in our center were included. We opted for a questionnaire with 3 variables: socio-demographics, clinical examination data, and the type of prosthetic treatment adopted. The study was conducted in the Department of Removable Prosthodontics in the Center of Consultation and Dental Treatment of Rabat Morocco (CCDTR) from September 2020 to July 2021.

**Results:**

the study population consisted of 91 patients, with a majority of male patients at 53% (n=48). During our study period, the proportion of patients under one-year-old was predominant, accounting for 38.4% (n=35) of the total sample. Among the patients in the sample, 72.5% (n=66) had no profession, while 66% (n=60) had a low income. Regarding medical insurance, the majority of our patients, 85.5% (n=78), were covered by Public Health Insurance for the Low-income Population (PHILP). Among the total sample, 57.1% (n=52) consulted for a combination of pain function and aesthetics. Additionally, 61.5% (n=56) were referred by teaching hospitals. For the type of oro-facial defect, 52.7% (n=48) of the sample consisted of newborns with congenital facial cleft. Out of the 91 patients, 36 had maxillofacial tumors, with 47.6% (n=17) of them presenting squamous cell carcinoma. Furthermore, 63.7% (n=58) of the prosthetic treatments adopted involved presurgical orthopedic treatments for newborns with facial cleft.

**Conclusion:**

the study on the epidemiological profile of patients attending the maxillofacial prosthodontics unit at Ibn Sina University Hospital in Rabat, Morocco provides important insights. The findings highlight the predominance of male patients and the prevalence of oro-facial defects in newborns. Socioeconomic factors, such as low income and lack of profession, are significant considerations. The majority of patients are covered by the PHILP, indicating the importance of medical insurance. These findings contribute to improving healthcare planning and specialized care for this patient population.

## Introduction

Maxillofacial prosthodontics (MFP) is a combination of the art and science of artificial reconstruction of the facial bone in cases of acquired substance loss or congenital malformations. The main objective of this discipline tends towards functional, aesthetic, and psychological rehabilitation. It is located at a crossroads between ORL, maxillo-facial surgery, and dental specialties [1,2].

Social integration and enhancement of the patient's quality of life are its main concerns. The cervico-facial reconstructive surgery development was didn't render MFP obsolete, on the contrary, their complementarity is now stronger than ever. In certain clinical situations the MFP can serve as a tissue conditioner for surgical procedures, and in other cases bring the final touches to help the patient regain optimal oro-facial functions. Surgical resections or adjuvant radiotherapy can have some undesirable side effects such as mouth opening, and tissue scars. These problems can be managed by the PMF as well [3,4].

The CCDTR is a care, research, and teaching center, providing dental care to Rabat's population and its surrounding areas. The maxillofacial prosthesis unit at this center is one of the two MFP units existing in Morocco, its specificity is based on the care of patients requiring rehabilitation by maxillofacial prostheses, such as maxillary obturator, dental prosthesis on a patient after mandibulectomy, facial epithesis, management of newborn with facial clefts, buccal management of patient before, during and after radiotherapy, sleep apnea management etc. It works in close collaboration with other medical and surgical specialists.

This study aimed to gain a clear understanding of the epidemiological profile of patients attending the maxillofacial prosthodontics unit at Ibn Sina University Hospital in Rabat, Morocco. The primary objectives were to identify the demographic, socioeconomic, and medical characteristics of the patient population, and to contribute to resource planning, targeted interventions, and scientific research in the field. The findings from this study provide valuable insights that can enhance healthcare planning and improve specialized care for this specific patient group.

## Methods

**Study design and setting:** this study was a cross-sectional study in which we collected data using structured questionnaires. The goal was to obtain information concerning socio-demographics, clinical examination data, and the type of prosthetic treatment adopted in the maxillofacial prosthodontics unit in Rabat, Morocco. The CCDTR is a care, research, and teaching center, providing dental care to Rabat's population and its surrounding areas. The maxillofacial prosthesis unit of the CCDTR is one of the two MFP units existing in Morocco, its specificity is based on the care of patients requiring rehabilitation by maxillofacial prosthesis.

**Study population:** the participants were all patients consulting the maxillo-facial prosthetics unit of Rabat, Morocco between September 2020 and July 2021 (11 months). During the timeline of our study, 91 patients were enrolled.

**Data collection:** structured questionnaires were distributed to patients or parents of newborns. The variables assessed in this study are Socio-demographic data: gender, age socio-economic level, the structure that referred the patient to our center, the patient's place of residence, and type of health insurance. Secondly, the variables related to the clinical examination: patient's general health status, patient motive for consultation, the diagnosis of the tumor for cancer patients, patient's type of loss of substance. The third variable concerns the type of prosthetic treatment adopted: maxillary obturator, mandibular prosthesis after mandibulectomy, facial epithesis, radiation therapy management, presurgical infant orthopedics, or pressotherapy for patients with facial scars caused by burns.

**Statistical analysis:** it was performed using the statistical package for social sciences SPSS version 19.0 software. The graphics were done in Microsoft Excel version 16.18 software.

**Difficulties and biases:** during this study, we encountered certain problems related to the lack of information in the patients' clinical files (histological examination, radio/chemotherapy session schedule). The non-cooperation of some patients, especially newborns and elderly patients with deteriorated health. Not having answers for certain questions related to the quality of life: mental/psychological state.

**Ethical consideration:** ethical clearance for the study was obtained from the Mohammed V University of Rabat Ethics Committee. Before data collection, participants of all ages with craniofacial defects were required to provide written informed consent. The consent form clearly outlined the study's objectives, procedures, and potential risks or benefits. Participants were informed of the voluntary nature of their participation and their right to withdraw without facing any consequences. For newborns and children, the consent process and study protocol were explained to their parents or legal guardians. Confidentiality was prioritized throughout the study, and participants were assured that their privacy and anonymity would be protected. The involvement of participants of all age groups was crucial in expanding our understanding of craniofacial conditions and enhancing future treatment approaches. To ensure participant anonymity, our questionnaire was meticulously designed. No personally identifiable information was collected or included in the questionnaire. Instead, data were coded or assigned unique identifiers to maintain confidentiality and protect anonymity. This approach enabled us to analyze the data in an aggregated and anonymous manner, safeguarding the identity of individual participants.

## Results

**Figure 1:**
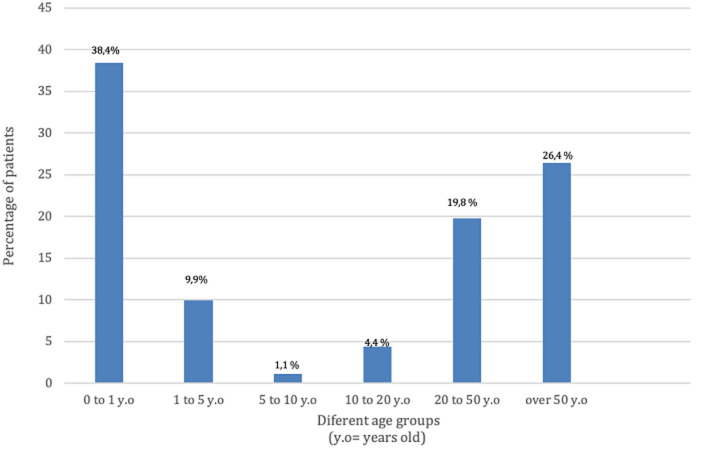
different age groups among patients consulting the maxillofacial prosthodontics unit in Rabat, Morocco

**Figure 2:**
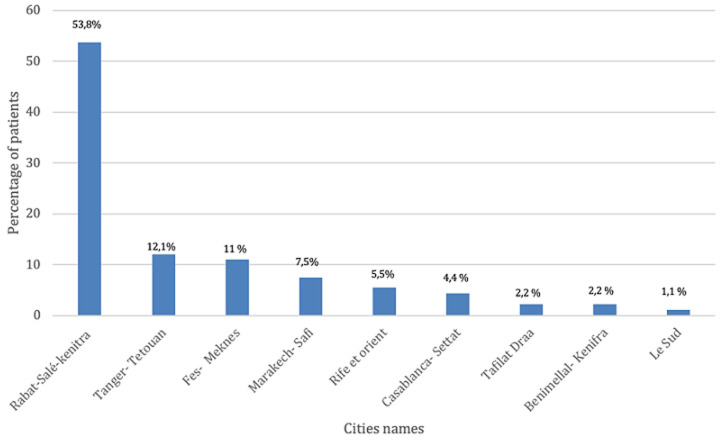
repartition of patients consulting the maxillofacial prosthodontics unit across different cities in Morocco

**Figure 3:**
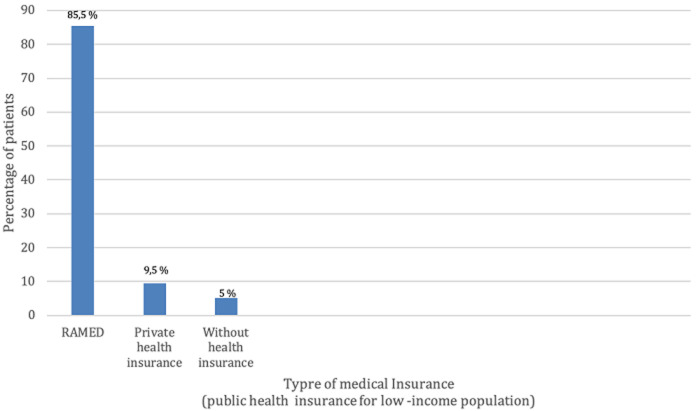
different types of health insurance among patients consulting the maxillofacial prosthodontics unit in Rabat, Morocco

**Socio-economic characteristics:** regarding gender, 53% (n=48) of patients are male and 47% (n=43) are female. The distribution according to age groups (Figure 1), showed that 38.4% (n=35) of patients are less than 1 year old, 26.4% (n=24) of our patients are over 50 years old, 19.8% (n=18) are between 20 and 50 years old, 9.9% (n=9) are between 1 and 5 years old, 4.4% (n=4) are between 10 and 20 years old, and 1.1% (n=1) are between 5 and 10 years old. Regarding our patients' place of residence (Figure 2), 53.8% (n=49) reside in the Rabat/Salé/Kenitra regions, 12.1% (n=11) reside in the Tangier/Tetouan region, 11% (n=10) reside in the Fes/Meknes region, 7.7% (n=7) reside in the Marrakech/Safi region, 5.5% (n=5) reside in the Rif/Orient region, 4.4% (n=4) reside in the Casablanca/Settat region, 2.2% (n=2) reside in the Draa/Tafilalet region, 2.2% (n=2) reside in the Khénifra /Béni melal region and 1.1% (n=1) of patients are residents of southern Morocco. The distribution according to the professional status showed that 72.5% (n=66) of the patients have no profession whereas 27.5% (n=25) of the patients have a profession. According to the socio-economic level we noted that 66% (n=60) of the patients have a low socioeconomic level, 32.8% (n=30) have an average socioeconomic level, and 1.2% (n=1) with a high socio-economic level. Regarding health insurance (Figure 3), 85.5% (n=78) of patients are covered with Public Health Insurance for the Low-income Population (PHILP), 9.5% (n=9) covered by private health insurance and 5% (n=4) are without medical insurance.

**Figure 4:**
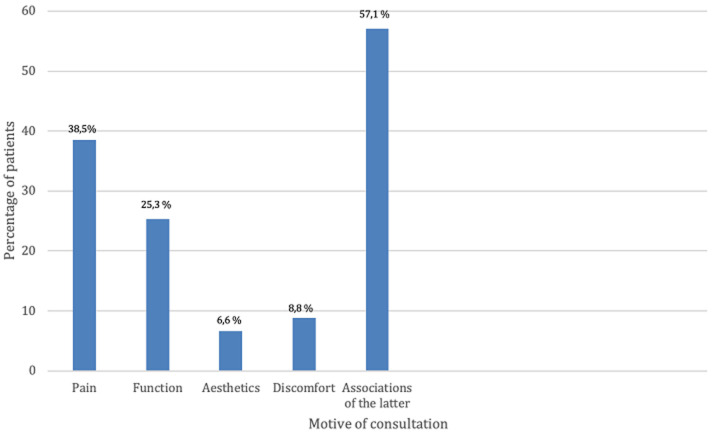
different motives of consultation in the maxillofacial prosthodontics unit in Rabat, Morocco

**Figure 5:**
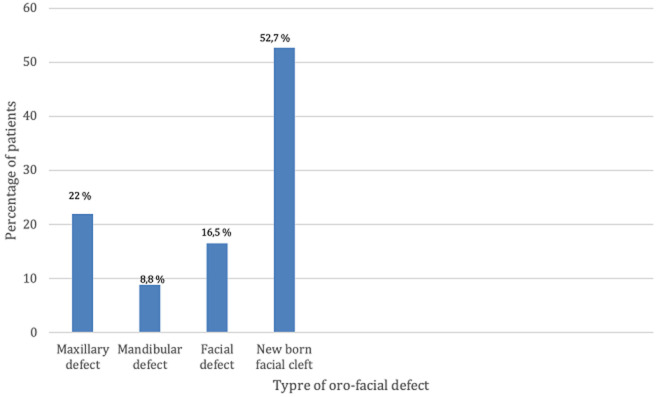
different types of oro-facial defects among patients consulting the maxillofacial prosthodontics unit in Rabat, Morocco

**Figure 6:**
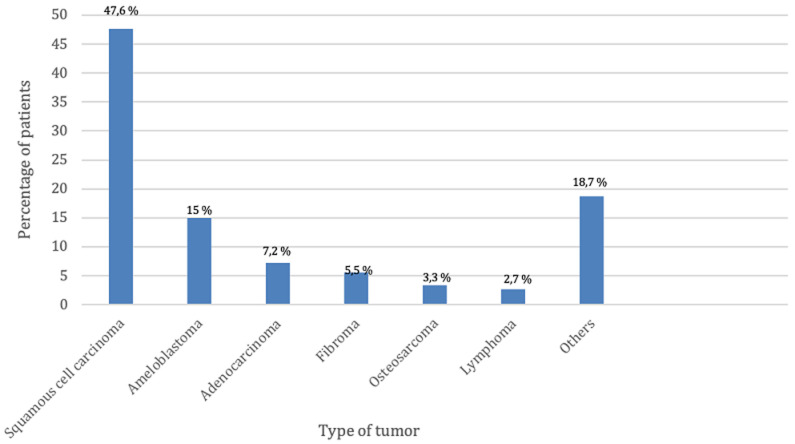
different tumor types among cancer patients consulting the maxillofacial prosthodontics unit in Rabat, Morocco

**Clinical profile:** we explored 4 questions which are: the patients' the motive of consultation, the referring hospital, the type of maxillofacial defect, and for cancer patients we asked the nature of the tumor. For the patients' motive of consultation (Figure 4), we noted that 57.1% (n=52) consulted for a combination of pain/function/aesthetical/and discomfort, 25.3% (n=23) for functional reasons, 8.8% (n=8) consulted because of discomfort, 6.6% (n=6) consulted for an aesthetical reason and 2.2% (n=2) of patients consulted for pain. Concerning the referring hospitals, we noted that 61.5% (n=56) of patients are referred by a teaching hospital, 20.9% (n=19) are referred by a peripheral hospital, and 17.6% (n=16) are referred by a private medical structure. According to the type of defect (Figure 5) we noted that 52.7% (n=48) of patients are newborn with congenital facial cleft, 22% (n=20) have maxillary defect, 16.5% (n=15) of patients have facial defect and 8.8% (n=8) of patients have mandibular defect. Regarding the type of tumor (Figure 6), out of 91 patients, 36 patients suffered a malignant or benign tumor among which 17 patients (47.6%) presented a squamous cell carcinoma, 5patients (15%) had an ameloblastoma, 4 patients (7.2%) presented an adenocarcinoma, 3 patients (5.5%) presented a fibroid tumor, 1 patient (3.5%) presented osteosarcoma, 1 patient (2.7%) presented a lymphoma and 6 patients (18.7%) presented with other lesion types.

**Figure 7:**
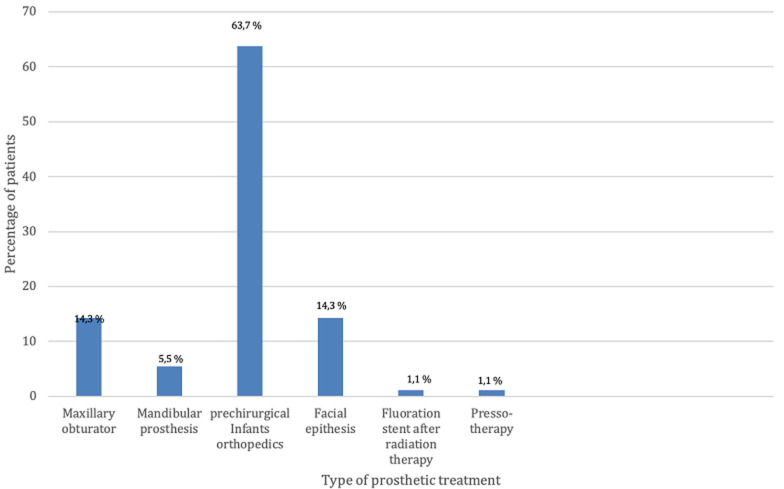
different types of prosthetic treatments received by patients consulting the maxillofacial prosthodontics unit in Rabat, Morocco

**Treatment and outcomes:** concerning the third variable of this study which is the type treatment adopted (Figure 7) among the 91 patients 63.7% (n=58) of patients required a pre-surgical infants orthopedics treatment, 14.3% (n=13) required maxillary obturator, 5.5% (n=5) of patients required mandibular prosthesis after mandibulectomy, 14.3% (n=13) of patients required facial epithesis, 1.1% (n=1) of patients require a fluoration stent after radiation therapy and 1.1% (n=1) of patients require pressotherapy to reduce facial scars caused by severe burns.

## Discussion

This study aimed to establish an epidemiological profile of patients consulting the unit of maxillofacial prosthodontics in Rabat. The results from this study are essential for enhancing the quality of patient care in our center and can also serve as a valuable comparison tool with other maxillofacial teams worldwide. During the 11-month study period, a total of 91 patients were included in the sample. The majority of patients were male, accounting for 53% of the sample. Notably, patients under one-year-old constituted the largest age group, making up 38.4% of the total sample. Socioeconomic factors played a crucial role, as 72.5% of the patients had no profession, and 66% had a low income. The majority of patients (85.5%) were covered by the PHILP in Morocco. The most common reasons for consultation were a combination of pain, function, and aesthetics, which accounted for 57.1% of the cases. Additionally, a significant number of patients (61.5%) were referred to the unit by teaching hospitals. Among the oro-facial defects, newborns with congenital facial clefts comprised the largest category, representing 52.7% of the sample. Maxillofacial tumors were present in 36 patients, with squamous cell carcinoma being the most frequent type (47.6%). Presurgical orthopedic treatments for newborns with facial clefts were the most commonly adopted prosthetic treatments, encompassing 63.7% of the cases.

Based on the socio-demographic profile encompassing age, sex, and socio-economic level, it is evident that the largest proportion of patients (38.5%) seeking consultation belongs to the category of newborns and children under one-year-old. This finding can be attributed to the higher prevalence of congenital etiologies and poly-malformation syndromes in this age group. These findings do not coincide with a study made by Mericske-Stern *et al*. [5]. They found that the majority of the patients consulting their center were adults over 50 with 26.4% with facial defects caused by tumors, probably because the incidence of cancers increases almost exponentially with age. According to our study, the male sex is predominant with a percentage of 53% compared to the female sex which is 47%, our results coincide with the literature. Many epidemiological studies showed that the incidence rate of cancers of the oral cavity is less common in women than in men, with a male/female incidence ratio of 6.9 [6]. The incidence of gender intervenes indirectly through the use of alcohol and tobacco [7]. Our results show that in general the socioeconomic level of the consulting population is low by 66%, this can be explained by the fact that 72.5% of our patients are unemployed and also the CCDTR is a public hospital, moreover, social health insurance covers many prosthodontics treatments, which is encouraging for Moroccan patients with low income to seek dental consultation in our center. Our results also coincide with the results of an epidemiological study carried out in Casablanca in 2014. It is mentioned as well in the literature, that nutritional status and socio-economic factors are closely linked to etiological factors in the occurrence of tumors and congenital deficits [8]. Indeed, disadvantaged populations or populations with a low or overall socioeconomic level are less medically monitored and therefore have less access to prevention measures and screening campaigns [9]. The same social class is more likely to adopt harmful lifestyle habits/lack of oral hygiene, and less balanced nutrition. According to our study, 53.8% of patients are from the Rabat-Sale-Kenitra region, while 46.2% of patients are from different regions with a predominance of the Tanger-Tetouan and Fes-Meknes regions, this is mainly explained by the fact that in Morocco outside of the major cities of Rabat and Casablanca there is lack or even the non-existence of specialist in maxillofacial prosthodontics. This shows that there is a real need for maxillofacial prosthodontics in the Moroccan population, and more centers need to be created in different regions to facilitate accessibility. Our results reveal that the majority of the consulting population is referred by teaching hospitals (in Rabat and Fes) 61.5% and that the rest of the patients are referred by a peripheral hospital 20.9% or a private medical structure 17.6%. This may be explained by the different levels of awareness of physicians, in fact in university hospitals, clinicians have access to updated information about the management of patients requiring a maxillo-facial treatment which is not necessarily the case with the other medical structures.

The main reason for consultation is an association of pain functions and aesthetic (57%) this is due to the impact of the loss of substance on the different functions of the orofacial sphere (food, phonation, breathing, articulation, and vision) as well as the acute (pain, mucositis) and chronic physical repercussions, mutilation, possible complications represented mainly by osteoradionecrosis, etc.), our results coincide with the results of the literature [10]. According to our study, the majority of consultations are due to congenital facial clefts on newborns, with a percentage of 53% which does not coincide with the results in the literature [11]. This may be explained by the geographical proximity of our center to the pediatric Hospital, also, the pediatric surgeons in Rabat have a better awareness of the different treatment options that our center offers. This percentage points out the necessity of establishing better communication with the maxillofacial surgeons and the oncologists in Rabat.

In comparing the results obtained from our study with those of the Brazilian study conducted in 2022 by Garcia *et al*. [12], several key points emerge. The Brazilian study had a larger sample size, consisting of 256 patients, compared to our study, which included 91 patients. This larger sample size in the Brazilian study may provide a more comprehensive representation of the subpopulation being studied. However, both studies focused on cancer patients requiring dental and oral-maxillofacial prostheses, indicating a common research interest. In the Brazilian study, 30.90% of the patients were older adults, while our study did not provide specific information about the age distribution of the patients. Additionally, the Brazilian study reported a higher proportion of male patients (65.6%), while our study had a slightly lower proportion (53%). These variations in demographic characteristics highlight potential differences in the patient populations under investigation. The Brazilian study focused on a specific subpopulation within Brazil, with 52% of the patients residing in municipalities in the state of Mato Grosso, excluding the capital. In contrast, our study was conducted in a different geographical location, namely Rabat and nearby areas. These regional differences may contribute to variations in patient demographics, disease prevalence, and treatment approaches. Both studies examined the types of oro-facial defects and tumor characteristics among the patient populations. The Brazilian study found that 57.4% of the tumors were located in the head and neck region, whereas our study did not provide specific information on tumor location. In terms of histological type, epidermoid carcinoma was the most frequent type in the Brazilian study (55.1%), while our study reported squamous cell carcinoma as the predominant type (47.6%) among the subset of patients with maxillofacial tumors. These differences in tumor characteristics highlight the potential variation in cancer profiles within different populations. Regarding prosthetic treatment, the Brazilian study reported that the majority of patients (60.2%) completed rehabilitation, primarily using total prostheses.

In contrast, our study revealed that presurgical infants' orthopedic treatments for newborns with facial clefts (63.7%) were the most common prosthetic treatment adopted. These differences in treatment approaches may reflect variations in the types of oro-facial defects and tumor characteristics prevalent in the respective populations. Finally, the Brazilian study provided valuable insights into the profile of cancer patients requiring dental and oral-maxillofacial prostheses in a specific subpopulation. Comparing our study with the Brazilian study, several differences were observed in terms of sample size, demographic characteristics, geographical factors, types of oro-facial defects, tumor characteristics, and treatment approaches. These differences emphasize the need for context-specific research to address the unique characteristics and challenges of different populations. Further studies and collaborations across different regions and populations can contribute to a more comprehensive understanding of the field and aid in the development of tailored interventions for patients requiring dental and oral-maxillofacial prostheses.

Furthermore, Quispe *et al*. [13], conducted a study involving 75 individuals, of which only 30 were cancer patients. The authors examined the need for maxillary and mandibular prostheses and reported the following findings: 21 patients required a maxillary prosthesis to replace one tooth (10%) or multiple teeth (33.3%), while some needed a combination of prostheses (13.3%) or a total prosthesis (13.7%). Among the participants, 29 individuals used a mandibular prosthesis to replace multiple teeth (70%), and a few required a combination of prostheses (3.3%) or a total prosthesis (23.7%).

Additionally, in the study titled “Maxillofacial prosthetic rehabilitation in the UK: a survey of maxillofacial prosthetists' and technologists' attitudes and opinions” conducted by Hatamleh *et al*. [14], the authors conducted a questionnaire survey among 220 maxillofacial prosthetists and technologists (MPTs) working in all UK maxillofacial units. The aim was to gather their opinions, attitudes, and experiences regarding various aspects related to maxillofacial silicone prostheses. The researchers analyzed the numbers and percentages of maxillofacial prostheses constructed, the methods of retention used, serviceability, causes of reduced serviceability, and the use of digital technologies (DT) in constructing the prostheses. A total of 1,193 prostheses were constructed, with 42% being ocular prostheses, 31% auricular prostheses, 13% orbital prostheses, 12% nasal prostheses, and 1% composite prostheses or more than one facial prosthesis.

During this study, we encountered certain challenges, including incomplete medical records and some non-cooperative patients. Nonetheless, our sample size was statistically sufficient to provide a reliable representation of the entire population. To minimize the risk of errors, we employed various measures, such as data processing software, a scientifically-based statistical method, and rigorous calculations to eliminate statistical errors. Despite the encountered difficulties and potential biases, their impact on the study was manageable and did not significantly distort our findings. Consequently, our study holds a satisfactory level of intrinsic value. However, it is essential to acknowledge that the external validity of our results is limited to the population consulting for maxillofacial prostheses between September 2020 and July 2021. Extending our findings beyond this specific period was not feasible due to the constraints of our study timeframe.

## Conclusion

This study provides valuable insights into the epidemiological profile of patients consulting the unit of maxillofacial prosthodontics at Ibn Sina University Hospital in Rabat, Morocco. The predominance of male patients and the higher proportion of newborns and children under one-year-old highlight important demographic trends. Socioeconomic factors, such as low income and lack of profession, were significant considerations among the patient population. The majority of patients were covered by the Moroccan Medical Assistance Scheme, underscoring the importance of low-income public health insurance. Oro-facial defects, especially congenital facial clefts, were prevalent, necessitating specialized care for this patient group. Maxillofacial tumors, with squamous cell carcinoma being the most frequent type, were also notable findings. The study's limitations should be acknowledged, such as incomplete medical files and non-cooperative patients. Despite these challenges, the study's findings contribute to improving healthcare planning and specialized care for patients with maxillofacial needs. However, any conclusions or recommendations beyond the data presented should be approached with caution, and further research is needed to address other aspects of patient care and potential developments in the field of maxillofacial prosthodontics.

### What is known about this topic

- *Maxillofacial prosthodontics is a specialized field of dentistry focused on restoring and improving oral and facial structures for patients with congenital or acquired defects;*
- *Oro-facial defects, such as congenital facial clefts and maxillofacial tumors, present significant challenges in patient care and require specialized treatment approaches;*
- *Socioeconomic factors play a crucial role in accessing healthcare services, especially for patients with maxillofacial needs, with public health insurance being an essential resource for many*.

### What this study adds

- *This study provides an epidemiological profile of patients consulting the unit of maxillofacial prosthodontics at Ibn Sina University Hospital in Rabat, Morocco, contributing valuable data on the patient population's characteristics and needs;*
- *The study highlights the predominance of male patients and a substantial proportion of newborns and children under one-year-old seeking maxillofacial care, emphasizing the significance of specialized pediatric maxillofacial prosthodontics care;*
- *Additionally, the prevalence of oro-facial defects, such as congenital facial clefts and maxillofacial tumors, underscores the importance of early diagnosis and specialized treatment planning for improved patient outcomes*.
